# A Semantic Model for Species Description Applied to the Ensign Wasps (Hymenoptera: Evaniidae) of New Caledonia

**DOI:** 10.1093/sysbio/syt028

**Published:** 2013-05-31

**Authors:** James P. Balhoff, István Mikó, Matthew J. Yoder, Patricia L. Mullins, Andrew R. Deans

**Affiliations:** ^1^National Evolutionary Synthesis Center, Durham, NC 27705, USA; ^2^Department of Biology, University of North Carolina, Chapel Hill, NC 27599, USA; ^3^Insect Museum, Department of Entomology, North Carolina State University, Box 7613, Raleigh, NC 27695, USA; ^4^Department of Entomology, Pennsylvania State University, 501 ASI Building, University Park, PA 16802, USA; ^5^Illinois Natural History Survey, University of Illinois, 1816 South Oak Street, MC 652 Champaign, IL 61820, USA; and ^6^Department of Ecology, Evolution, and Organismal Biology, Iowa State University, Ames, IA 50011, USA

## Abstract

Taxonomic descriptions are unparalleled sources of knowledge of life's phenotypic diversity. As natural language prose, these data sets are largely refractory to computation and integration with other sources of phenotypic data. By formalizing taxonomic descriptions using ontology-based semantic representation, we aim to increase the reusability and computability of taxonomists' primary data. Here, we present a revision of the ensign wasp (Hymenoptera: Evaniidae) fauna of New Caledonia using this new model for species description. Descriptive matrices, specimen data, and taxonomic nomenclature are gathered in a unified Web-based application, mx, then exported as both traditional taxonomic treatments and semantic statements using the OWL Web Ontology Language. Character:character-state combinations are then annotated following the entity–quality phenotype model, originally developed to represent mutant model organism phenotype data; concepts of anatomy are drawn from the Hymenoptera Anatomy Ontology and linked to phenotype descriptors from the Phenotypic Quality Ontology. The resulting set of semantic statements is provided in Resource Description Framework format. Applying the model to real data, that is, specimens, taxonomic names, diagnoses, descriptions, and redescriptions, provides us with a foundation to discuss limitations and potential benefits such as automated data integration and reasoner-driven queries. Four species of ensign wasp are now known to occur in New Caledonia: *Szepligetella levipetiolata*, *Szepligetella deercreeki* Deans and Mikó sp. nov., *Szepligetella irwini* Deans and Mikó sp. nov., and the nearly cosmopolitan *Evania appendigaster*. A fifth species, *Szepligetella sericea*, including *Szepligetella impressa*, syn. nov., has not yet been collected in New Caledonia but can be found on islands throughout the Pacific and so is included in the diagnostic key. [Biodiversity informatics; Evaniidae; New Caledonia; new species; ontology; semantic phenotypes; semantic species description; taxonomy.]

Taxonomic descriptions constitute an invaluable source of knowledge about phenotypic diversity across the living world. However, these phenomic annotations are not readily accessed and reused by other biological scientists (Deans et al. 2012). Instead, they are “locked away” in the taxonomic literature, written for, and consumed almost exclusively by, taxonomists. While electronic availability of taxonomic treatments is rapidly growing, reflected in changes to publication requirements (International Commission on Zoological Nomenclature 2012), and accelerated by digitization efforts such as the Biodiversity Heritage Library (http://www.biodiversitylibrary.org/ last accessed May 13, 2013), the constituent phenotypic descriptions are composed in natural language (NL), typically making use of specialized anatomical terminology. These phenotypic descriptions are difficult to data-mine (though see Cui (2012); Thessen et al. (2012)); one reason for this is rampant homonymy and synonymy across anatomical concepts (Yoder et al. 2010). We recently proposed that the application of ontological annotation to taxonomic descriptions, as semantic phenotypes (SPs), would allow a broader array of researchers to apply powerful data integration, search, and automated reasoning techniques to these data, increasing the value of taxonomic work and promoting its reuse (Deans et al. 2012).

An ontology is a formal representation of concepts within a domain and the logical relationships between those concepts, supporting knowledge representation with explicit semantics. By referencing standard, shared concepts, diverse data sets can be aggregated and computed over coherently (Washington et al. 2009; Walls et al. 2012). Biological ontologies have become a standard tool for organizing and accessing genomic and phenotypic data from taxonomically isolated model species (Mungall et al. 2010). Applying these tools to the representation and dissemination of comparative descriptive data offers a means to make connections of phenotypic and genomic information across these different, but closely related, sciences (Mabee et al. 2007; Deans et al. 2012).

The Phenoscape project has pioneered the application of ontological annotation to evolutionary phenotypes, by annotating morphological character matrix data sets from the published fish systematics literature (Dahdul et al. 2010a), and demonstrating semantic correspondences to mutant phenotype annotations from the Zebrafish Information Network, ZFIN (Mabee et al. 2012). We believe that taxonomists can build on this approach by incorporating ontological annotation into descriptions at the time of publication, thereby increasing the efficiency and scalability of SP annotation. As taxonomists adopt this approach, tools may be developed that facilitate referencing and integrating with existing semantic data as part of the process of creating new descriptions. Here, we discuss our first steps toward meeting this goal by describing a model for SPs, by using the model with real data, and by discussing directions for further advancing these methods. Our current approach to integrating semantic components with taxonomic treatise is to provide a “traditional” NL description in parallel with a set of semantic annotations. These annotations are made possible by the advent of a relatively new data construct, the multispecies anatomy ontology, which contains generalized semantic definitions for anatomical concepts across a broad taxonomic group (Dahdul et al. 2010b). Annotations (SPs) are logically composed using references to these anatomical concepts, in conjunction with descriptive concepts from other ontologies such as PATO, an ontology of phenotypic descriptors (Mungall et al. 2010), and BSPO, an ontology of biospatial terms, for example, “proximal” and “anterior” (Table 1).

**Table 1.** Ontologies referenced in the SPs described here

Ontology	URI	Use
Hymenoptera Anatomy Ontology (HAO)	http://purl.obolibrary.org/obo/hao.owl	Anatomy of hymenopterans, e.g., “seta”, “mesosoma”.
Phenotype and Trait Ontology (PATO)	http://purl.obolibrary.org/obo/pato.owl	Phenotypic qualities, e.g., “blue”, “foveate”, “sigmoid”.
Biospatial Ontology (BSPO)	http://purl.obolibrary.org/obo/bspo.owl	Specification of spatial regions within anatomical parts, e.g., “lateral region”, “anterior margin”.
Comparative Data Analysis Ontology (CDAO)	http://purl.obolibrary.org/obo/cdao.owl	Data matrix elements, e.g., “Standard Character”, “TU”.
Darwin-SW	http://purl.org/dsw/	Specimen metadata, e.g., “Specimen”, “Identification”.
Information Artifact Ontology (IAO)	http://purl.obolibrary.org/obo/iao.owl	Information entities, e.g., “denotes” property.
Relations Ontology (RO)	http://purl.obolibrary.org/obo/ro.owl	Standard property definitions, e.g., “part of”, “is bearer of”.

Here, we demonstrate the application of SPs to the taxonomy of ensign wasps (Evaniidae). By working with real data early in the development of methodologies employing SPs, we identify and address some of the limitations of this approach. We conclude with a discussion as to both the real and perceived future problems of SPs as used here and highlight potential explorations that may advance the approach in subsequent work.

Ensign wasps are charismatic hymenopterans that develop as solitary predators of cockroach (Blattodea) eggs inside oothecae (Deans 2005). Despite their ubiquity in the tropics and ease of diagnosis at the family and genus level, very little is known of ensign wasp behavior, host associations, ecology, and other aspects of their biology. This situation is largely the result of an unsystematic species-level taxonomy and the paucity of tools for identification. In this article, we revise the ensign wasp fauna of New Caledonia, a relatively remote island in the South Pacific that is celebrated as a biodiversity hotspot with extraordinarily high levels of endemism [~80% of plant species found in New Caledonia are endemic (Morat 1993; Jaffré et al. 2001)] and microendemism (intraisland endemism; see Murienne et al. 2005). Given the size of this landmass, its geologic history, and its unique flora and fauna, New Caledonia has served as a laboratory for testing hypotheses about biogeography and speciation. Several of these studies have focused on cockroaches (Murienne et al. 2005, 2008), which will undoubtedly be relevant to future, finer-scale studies aimed at understanding the Evaniidae of New Caledonia. Deans (2005) cataloged the world ensign wasp fauna, and Deans et al. (2006) provided the first phylogenetic estimation of generic relationships. These two publications serve as the foundation for an active program in ensign wasp systematics which will hopefully remove roadblocks to future ensign wasp research (e.g., Kawada and Azevedo 2007; Deans and Kawada 2008; Mullins et al. 2012). Our current focus is to provide an updated classification for this fauna and to provide the tools necessary for species-level diagnosis.

## Methods

### Modeling SPs

Our SPs follow the entity–quality (EQ) approach, meaning we draw “entity” terms from an organism-specific anatomy ontology, and phenotypic quality terms from a taxon-agnostic ontology of qualitative descriptors. EQ is a guiding principle for formulating ontological class expressions (SPs), which represent the class of organisms that a given character state denotes. The phenotype class expressions we created follow the approach advocated by Mungall et al. (2007), which we extended to generally adhere to four “template” structures, and to draw on a rigorous classification of morphological characters (Sereno 2007) which is consistent with EQ (Dahdul et al. 2010a). For this study, entity terms were drawn from the Hymenoptera Anatomy Ontology (HAO) and the Biospatial Ontology (BSPO), whereas quality descriptors were drawn from the Phenotype and Trait Ontology (PATO) (Table 1). The full data set, represented in OWL (Web Ontology Language; http://www.w3.org/TR/owl2-overview/ last accessed May 13, 2013), was deposited as an Resource Description Framework (RDF)-XML file (http://www.w3.org/TR/REC-rdf-syntax/ last accessed May 13, 2013) in the Dryad repository (http://dx.doi.org/10.5061/dryad.2gd84 last accessed May 13, 2013).

### Observations

Diagnostic characters were discovered during direct examination of specimens under an Olympus SZX16 stereomicroscope and by comparing standard view images of specimens. Digital images were made using an Olympus CX41 compound microscope, equipped with a DP71 digital camera. SEM micrographs were taken by Philips XL30 ESEM-FEG (ISU) and FEI Nova 400 NanoSEM (FSU) on Au–Pd-coated specimens. Original images are deposited at Morphbank (http://morphbank.net last accessed May 13, 2013), as image collection 783132. Verbatim specimen label data and museum coden information are provided in online Appendix 2. Taxonomic nomenclature, specimen data, supporting images, and character matrix-based descriptive statements were compiled in the open-source web application mx (http://purl.org/NET/mx-database last accessed May 13, 2013) through interactive forms and integrated batch-uploading. NL treatments that include nomenclatural, descriptive, and material-related sections were rendered from these data using automated mechanisms included in mx. NL character descriptions were formulated with ontology annotation in mind, and in some cases revised to facilitate annotation.

### Generating SPs

**Figure 1:**
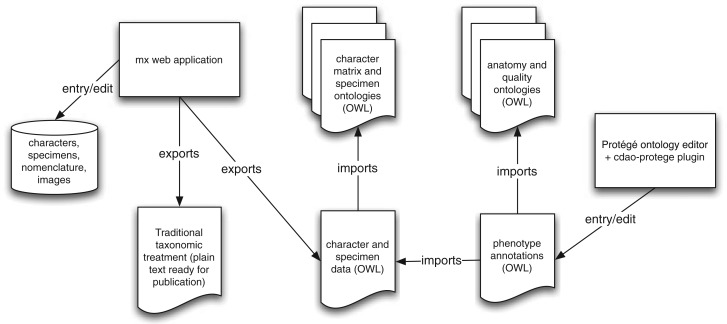
Workflow for data entry and semantic annotation. Observational and nomenclatural data are edited and stored within the mx web application. These data can be exported as NL descriptions for publication, or as structured data in OWL format. The OWL-formatted description data are referenced within SP annotations entered, using Protégé, into a standalone OWL document.

Once the data for the complete set of taxonomic treatments were captured within mx, the descriptive matrix elements and specimen identifiers were exported to OWL, using functions newly developed for this purpose within the mx application (Fig. 1). The OWL-formatted descriptive data were represented using terms from the Comparative Data Analysis Ontology (CDAO) (Prosdocimi et al. 2009) and Darwin-SW, an ontology of Darwin Core terms (http://code.google.com/p/darwin-sw/ last accessed May 13, 2013). These data, along with ontologies listed in Table 1, were loaded into Protégé 4.1 (http://protege.stanford.edu/ last accessed May 13, 2013). Navigation of the descriptive matrix elements in Protégé was aided by a custom-built plugin, available from the Github source code repository (https://github.com/balhoff/cdao-protege last accessed May 13, 2013). SP annotations were added to character states within Protégé as OWL class expressions using the built-in Manchester syntax (http://www.w3.org/TR/owl2-manchester-syntax/) last accessed May 13, 2013 editor. These phenotype annotation axioms were created in a separate OWL file which imported the character matrix OWL file exported from mx; this allowed edits to character data within mx to be integrated with in-progress phenotype annotation work in Protégé, by re-exporting and replacing the character matrix file (Fig. 1).

### Querying the OWL Data Set

Summary queries over the OWL-annotated data set were performed using custom programs written in Scala, using the OWL API programming library, version 3.2.4 (http://owlapi.sourceforge.net/ last accessed May 13, 2013) and the FaCT++ Description Logic reasoner, version 1.5.3 (http://code.google.com/p/factplusplus/ last accessed May 13, 2013). We performed the same queries over a comparison data set covering another ensign wasp genus, generated in parallel to this one (Mullins et al. 2012).

## Results

### SP Model

The SP expressions we created build on the OWL representation for EQ explicated by Mungall et al. (2007). In this article, all ontological expressions will be presented using the OWL Manchester syntax, a user-friendly but precise syntax for OWL 2 descriptions (http://www.w3.org/TR/owl2-manchester-syntax/ last accessed May 13, 2013). For example, the “some” keyword in the following expressions signifies an existentially quantified property restriction. For a basic phenotype such as “wing shape: curved,” Mungall et al. (2007) demonstrate two cognate forms of OWL phenotype expressions: those described from the perspective of the quality, for example, “curved and *inheres_in* some wing” (their so-called “normal-form”), and those described from the perspective of the entity, for example, “wing and *bearer_of* some curved.” We adopted the entity-based form, which can be more conveniently associated with a specimen exhibiting the phenotype, through a *has_part* relationship:


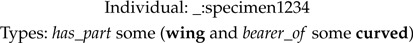


It is not necessary to explicitly include “shape” in the expression; the knowledge that “curved” is a subtype of “shape” is built into the PATO ontology.

In many cases, the structure that bears the quality being described is an instance of a “general” class, which must be further specified. For example, this may be a class of repeated anatomical structure, such as “bristle,” or a class denoting an abstract spatial region; both of these require localization to a specific, containing, anatomical structure, such as “mesosoma.” In the terminology of Sereno (2007), for a character such as “ventro-lateral region of mesosoma texture: foveate,” the entity bearing the quality (here, ventro-lateral region) is the *primary locator*, whereas the containing structure is a *secondary locator* (here, mesosoma). We used a nested series of *has_part* restrictions, mapping neatly to Sereno's secondary locator(s), L:


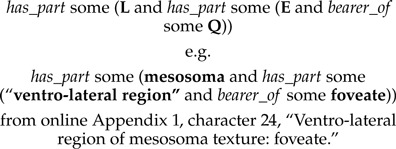


Context-dependent anatomical entities such as the above are often described as standalone expressions using a “post-composition” approach in other EQ annotation software, such as Phenex (Balhoff et al. 2010). The entity portion of the EQ might be described as an instance of a class such as:


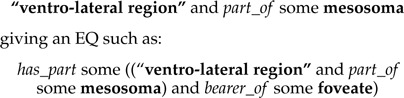


However, we found that postcompositions in our annotations were nearly always used to express parthood relationships; these structures could instead be represented using the aforementioned *has_part* chain, which provides two advantages over the *part_of* construction: (i) the entity class is more proximately associated with the quality it bears within the Manchester syntax expression, helping the human annotator verify correctness of the expression and (ii) an automated reasoner can infer that the “locator” structure is part of the same organism as the entity structure, a fact that is not implied by the semantics of the *part_of* -based class expression.

Building upon the basic EQ construct just described, we identified four template EQ expressions which could be used to express the meaning of the various character forms in the matrix:


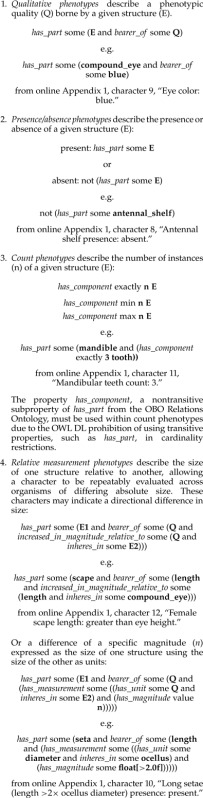


Relative measurement phenotypes highlighted an important limitation of OWL class expressions: without the ability to include variables within the expression, it was impossible to fully represent the intended meaning (Motik et al. 2009). Namely, a critical aspect of the phenotype is that the two structures being compared are components of the very same organism or containing structure. For a phenotype such as “antenna longer than eye,” we might create the following class expression:


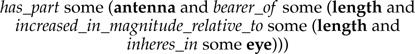


Unsatisfyingly, to be an instance of this class, an antenna needs to merely be longer than at least one eye in the world, not necessarily an eye possessed by the same organism. Although the semantics of the above phenotype description are not complete with respect to the meaning of the character, they still provide useful information about character data and specimens by making it clear that the given character describes aspects of antenna and eye size. Indeed, for not only relative measurement phenotypes but in fact all phenotype descriptions we created, the OWL class representing a phenotype for a given character state is defined not as equivalent to the EQ description but rather as a subclass—the provided semantics are necessary aspects of the phenotype but not a wholly sufficient description. To make the intended semantics of relative measurement phenotypes more explicit to consumers of the semantic description, we added a rule for each such character state using Semantic Web Rule Language (http://www.w3.org/Submission/SWRL/ last accessed May 13, 2013). For the example phenotype above, the corresponding rule would state that if there is an organism which has as parts an antenna and an eye, and the antenna is *increased_in_magnitude_relative_to* the eye, then that organism is inferred to be a member of the class defining the phenotype. Within our data set, these rules are not actually exercised; they are included only as a clarification of relative measurement semantics within the limitations of the OWL 2 language.

Of the 43 annotated characters, 24 fell into the qualitative category whereas 13 described a presence/absence. Only one described a count, whereas five described relative measurements. Only four characters (nos. 14, 38, 39, and 43) significantly departed from the basic templates, usually by incorporating more complicated intersection or union expressions. All SP expressions along with the original natural-language characters can be found in online Appendix 1.

### Linking SPs to Character Data and Specimens

A phenotype annotation consists of an OWL class, P, the ontological description of that class using an EQ expression, and the linkage of the phenotype class to a particular character state, CS, by means of a class assertion:

CS Type (*denotes* only **P**)

The *denotes* property is defined within the Information Artifact Ontology (IAO) to signify a reference by an informational entity to a “portion of reality.”

**Figure 2:**
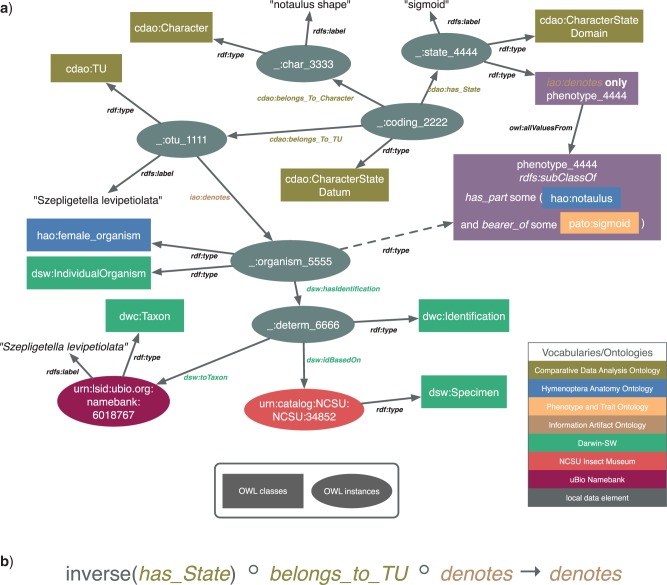
An OWL/RDF model showing explicit semantic links between natural-language character matrix data, an ontological phenotype representation, and a museum specimen with taxonomic metadata. In (a), a character matrix cell (_:coding_2222) is represented using the CDAO, upper half, linked to a museum specimen (urn:catalog:NCSU:NCSU:34852) described with the Darwin-SW ontology for Darwin Core, lower half. An EQ representation of the phenotype denoted by the given character state has been composed using terms from the HAO and the PATO. The *denotes* property, from the IAO, is used to bridge observational data artifacts (CDAO data elements) to direct descriptions of organisms (as EQ phenotypes). By applying an OWL 2 DL reasoner to the character matrix model, we can infer phenotypic characteristics of associated specimens (dashed arrow) using an OWL property chain (b).

We made use of *denotes* to connect both the character states and the Operational Taxonomic Units (OTUs) within a character matrix to the actual organisms (specimens) being described. So, since in our data model an OTU *denotes* a particular set of specimens under investigation, it follows that a character state *denotes* any specimen whose OTU has that state as a matrix value. We encoded this assertion by defining an OWL property chain (a property chain describes a path of links in the RDF graph which imply a new direct link between the nodes at either end of the path) (Fig. 2). By connecting phenotype classes to character states using a universally quantified restriction (*denotes* only **P**), we can infer that any specimens denoted by OTUs possessing a given character state (and thus denoted also by that character state) are members of that phenotype class. This logical framework allows us to propagate phenotypes to specimens while directly asserting semantic annotations only for character states (Fig. 2).

### Ontology Concept Usage

As preliminary examples for how ontology-based annotation facilitates cross-data set computation, we performed queries, programmed using the OWL API, over both this data set and a comparison data set for a relatively distantly related genus, *Evaniscus* (Mullins et al. 2012). Because the two data sets make use of shared, community reference ontologies, we were able to directly compare concept (i.e., OWL class) usage across the two studies (Table 2). Although the two taxonomic descriptions of wasp genera were conducted by members of the same research group, the number of referenced ontology classes found in both studies was small relative to the total: 22 out of 100 hymenopteran anatomy concepts, 16 out of 54 quality descriptors, and only 4 out of 24 biospatial concepts. The *Szepligetella* data set (this study) referenced a markedly broader range of quality descriptors than the *Evaniscus* data set: 41 concepts across 43 characters versus 30 concepts across 56 characters.

**Table 2.** Counts of OWL classes used within EQ phenotype expressions, in this study, *Szepligetella*, versus Mullins et al. (2012), *Evaniscus*

Study	HAO	PATO	BSPO
*Szepligetella* (43 characters)	58	41	16
*Evaniscus* (56 characters)	64	30	12
Total (union)	100	54	24
Common (intersection)	22	16	4

“Common” classes are used in both studies.

We also used the ontologies to assess the distribution of study characters across selected anatomical and qualitative partitions (Table 3). By means of an automated reasoner (FaCT++, driven by our OWL API-based scripts), we queried for characters describing any structure known to be part of the region of interest, for example, “head.” As expected, based on our reading of current and past hymenopteran taxonomy literature, a large proportion of the characters in both studies concern features of the head, thorax, and integument. Although comparable proportions of characters in both studies described types of shape, size, and, to a lesser extent, texture, within the *Evaniscus* data set characters describing color variation are much more prevalent than in this study (20% vs. 5% of all characters).

**Table 3.** Percentage of characters annotated with EQ phenotype expressions involving the given class, in this study, *Szepligitella*, versus Mullins et al. (2012), *Evaniscus*

Ontology class*^a^*	*Szepligetella* (43 characters) (%)	*Evaniscus* (56 characters) (%)
integument (HAO:0000421)	63	46
head (HAO:0000397)	21	27
mouthparts (HAO:0000639)	2	4
genitalia (HAO:0000374)	0	0
mesosoma (HAO:0000576)	53	54
metasoma (HAO:0000626)	0	2
abdomen (HAO:0000015)	12	5
“sense organ” and part_of some “appendage” (HAO:0000930, HAO:0000144)	0	2
color (PATO:0000014)	5	20
shape (PATO:0000052)	28	21
size (PATO:0000117)	16	18
texture (PATO:0000150)	14	25

*^a^*For entities (HAO), characters are counted if they describe a phenotype subsumed by “(has_part some E) or (has_part some (part_of some E),” for entity E. For qualities (PATO), characters are counted if they describe a phenotype subsumed by “has_part some (bearer_of some Q),” for quality Q.

## Identification Key for New Caledonian Evaniidae

**Figure 3:**
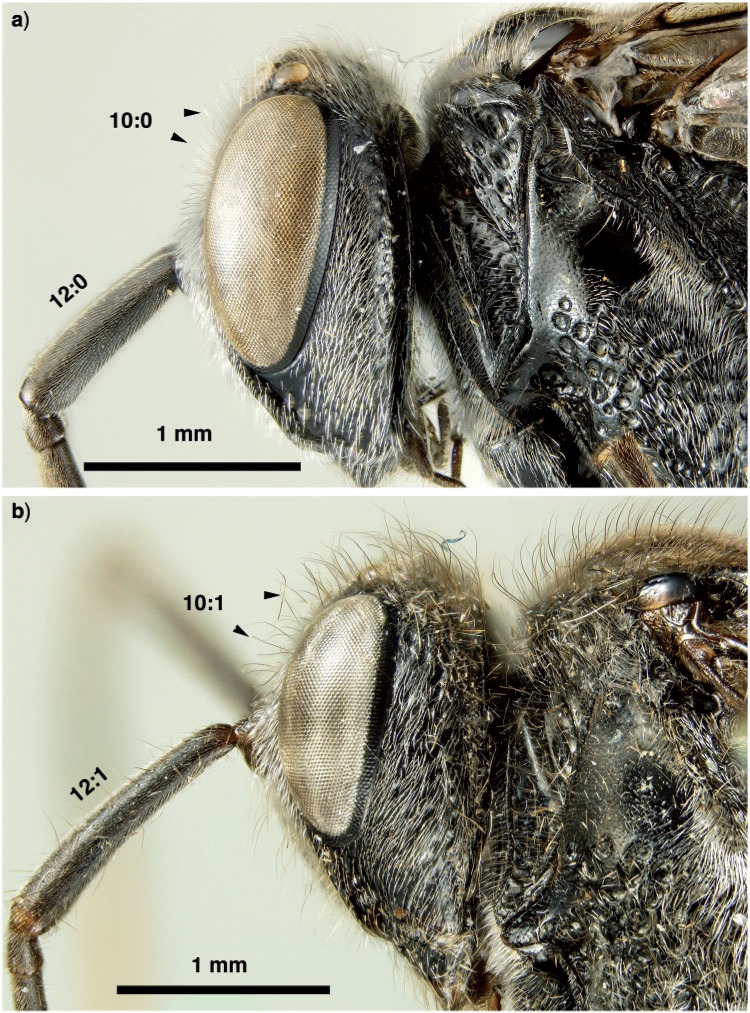
Brightfield images of the head and anterior thorax in New Caledonian *Szepligetella* species, lateral view. a) *Szepligetella deercreeki* Deans and Mikó sp. nov. b) *Szepligetella irwini* Deans and Mikó sp. nov. presence: absent (Fig. 7e) . . . *Szepligetella sericea* (Cameron 1883).

**Figure 4:**
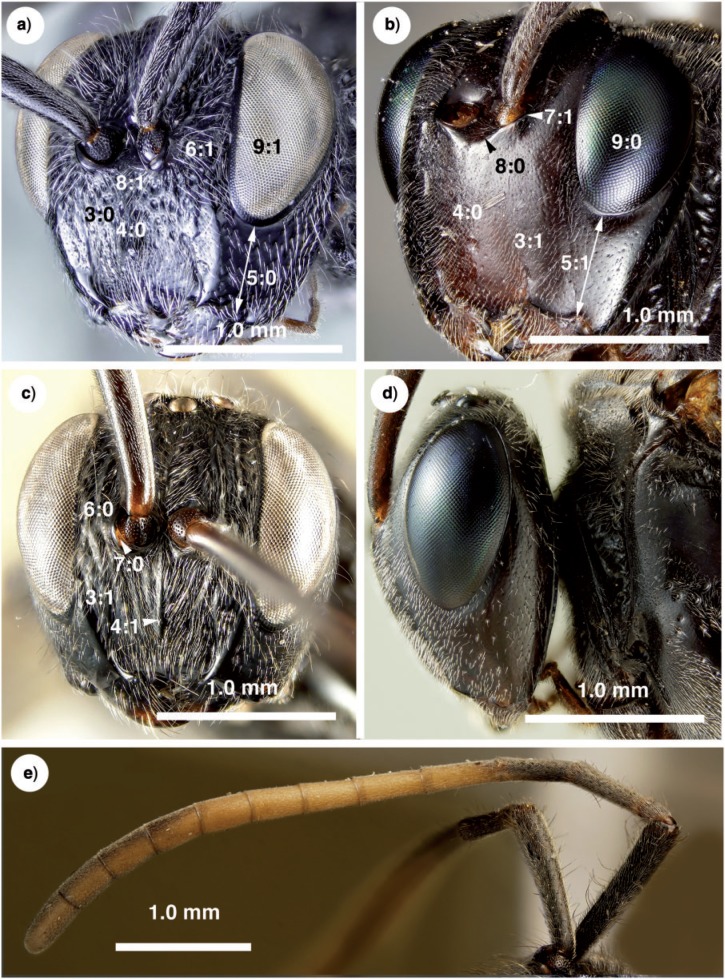
Brightfield images of the head and antenna in New Caledonian Evaniidae. a) *Szepligetella deercreeki* Deans and Mikó sp. nov., head, anterolateral view. b) *Evania appendigaster* (Linneaus 1758), head, anterolateral view. c) *Szepligetella levipetiolata* (Turner 1919), head, anterior view. d) *Evania appendigaster* (Linneaus 1758), head, lateral view. e) *Szepligetella irwini* Deans and Mikó sp. nov., antenna, lateral view.

1. Antennal shelf presence: present (Fig. 4b); eye color: blue (Fig. 4b); malar space length: longer than 0.5 of compound eye height (Fig. 4b); ventro-lateral region of mesosoma texture: foveate (Fig. 6c); ventral margin of mesopectus length: shorter than ventral margin of metapectus length (Fig. 6c) . . . *Evania appendigaster* (Linnaeus 1758).
  – Antennal shelf presence: absent (Fig. 4a); eye color: gray-silver (Fig. 4a); malar space length: shorter than 0.5 of compound eye height (Fig. 4a); ventro-lateral region of mesosoma texture: areolate (Fig. 6a); ventral margin of mesopectus length: longer than ventral margin of metapectus length (Fig. 6a).
2. Female flagellum color pattern: banded (Figs. 4e and 6f); Carinae laterally on frons presence: present (Fig. 4c); lower face texture: foveae absent (Fig. 4b,c); female scape length: greater than eye height (Fig. 3b); female flagellum ventral sensillar patch (Fig. 3b) spatial arrangement: F6–F11; submedian propodeal projection presence: present (Fig. 6a,b).
  – Female flagellum color pattern: monocolored; Carinae laterally on frons presence: absent (Fig. 4a); lower face texture: foveate (Fig. 4a); female scape length: equal to eye height (Fig. 3a); female flagellum ventral sensillar patch (Fig. 3b) spatial arrangement: F4–F11; submedian propodeal projection presence: absent (Figs. 6c,e).
3. Long setae (length *>*2× ocellus diameter) presence: present (Fig. 3b); female metatibial spines presence: absent (Fig. 7b) . . . *Szepligetella irwini* Deans and Mikó sp. nov.
  – Long setae (length *>*2× ocellus diameter) presence: absent (Fig. 3a); female metatibial spines presence: present (Fig. 7a) . . . *Szepligetella levipetiolata* (Turner 1919).
4. Notaulus shape: falciform (Fig. 6d); notaulus lateral margins alignment: paralell (Fig. 6b,d); median mesoscutal area shape: not prominent relative to lateral mesoscutal area (Fig. 6b,d); lateral carina of gastral scrobe presence: present (Fig. 8d); petiole texture: furrowed (Fig. 7d); setiferous patch on dorsal region of female abdominal terga 4–7
  – Notaulus shape: sigmoid (Fig. 6a,b). Notaulus lateral margins alignment: diverging (Fig. 6e). Median mesoscutal area shape: prominent relative to lateral mesoscutal area (Fig. 6e). Lateral carina of gastral scrobe presence: absent (Fig. 8c,e). Petiole texture: smooth (Fig. 7c,e). Setiferous patch on dorsal region of female abdominal terga 4–7 presence: present (Fig. 7c) . . . *Szepligetella deercreeki* Deans and Mikó sp. nov.

## Taxonomic Treatment

**Figure 5:**
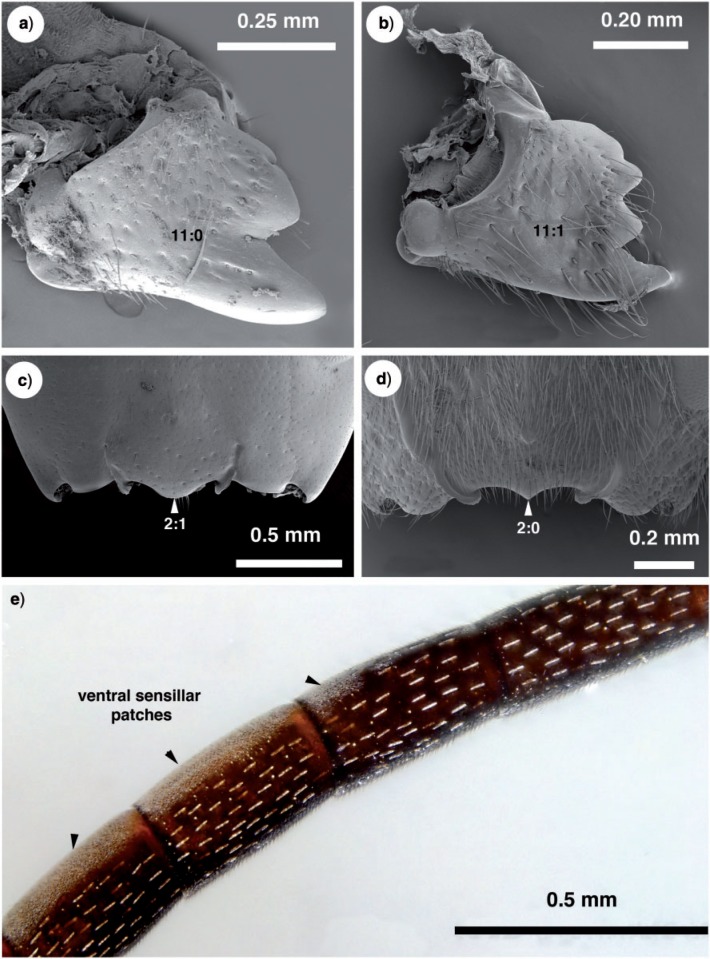
Brightfield images and SEM micrographs of the mandible, head capsule and flagellomeres in New Caledonian Evaniidae. a) *Evania appendigaster* (Linneaus 1758), right mandible, anterior (ventral) view. b) *Szepligetella levipetiolata* (Turner 1919), right mandible, anterior (ventral) view. c) *Evania appendigaster* (Linneaus 1758), ventral region of head capsule. d) *Szepligetella levipetiolata* (Turner 1919), ventral region of head capsule. e) *Evania appendigaster* (Linneaus 1758), flagellomeres 4–7.

**Figure 6:**
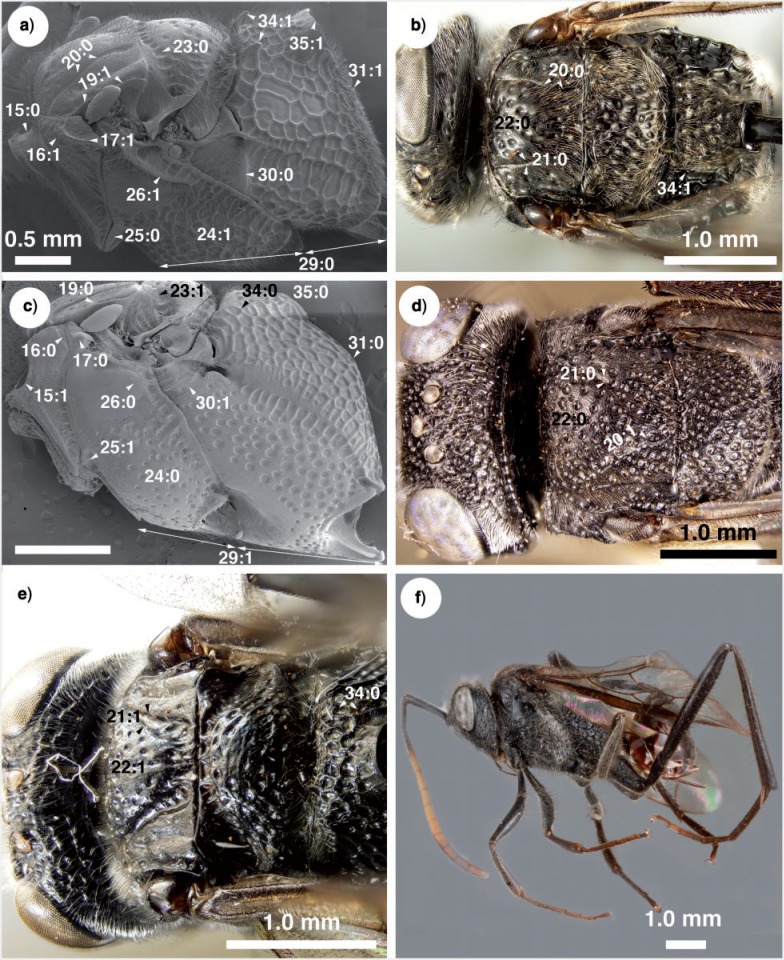
Brightfield images and SEM micrographs of New Caledonian Evaniidae. a) SEM micrograph of *Szepligetella levipetiolata* (Turner 1919), mesosoma, lateral view. b) *Szepligetella levipetiolata* (Turner 1919), head and mesosoma, dorsal view. c) *Evania apendigaster* (Linneaus 1758), mesosoma, lateral view. d) *Szepligetella sericea* (Cameron 1883). e) *Szepligetella deercreeki* Deans and Mikó sp. nov., head and mesosoma, posterodorsal view. f) *Szepligetella irwini* Deans and Mikó sp. nov., habitus, lateral view.

**Figure 7:**
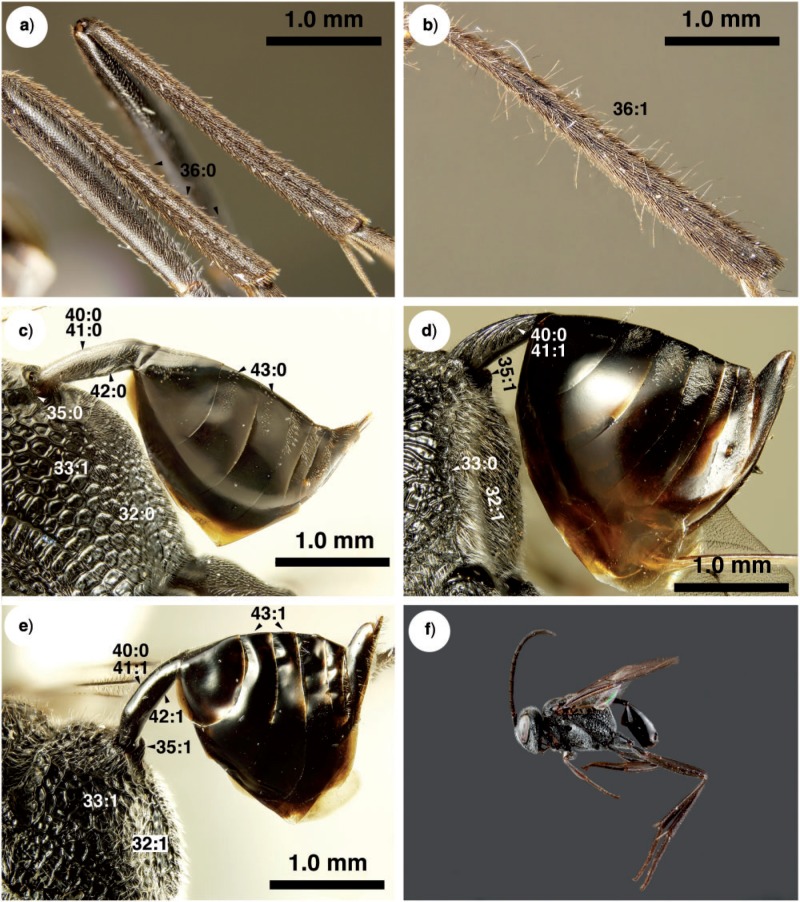
Brightfield images of New caledonian Evaniidae. a) *Szepligetella levipetiolata* (Turner 1919), left mid and hind tibiae and femora, lateral view. b) *Szepligetella irwini* Deans and Mikó sp. nov., left mid and hind tibiae and femora, lateral view. c) *Evania appendigaster* (Linneaus 1758), metapectal–propodeal complex and metasoma, posterolateral view. d) *Szepligetella sericea* (Cameron 1883), metapectal–propodeal complex and metasoma, posterolateral view. e) *Szepligetella deercreeki* Deans and Mikó sp. nov., metapectal–propodeal complex and metasoma, posterolateral view. f) *Szepligetella deercreeki* Deans and Mikó sp. nov., habitus, lateral view.

Evania appendigaster (*Linnaeus 1758) (Figs. 4b, d, 5a, c, e, 6c, 7c, 9c, d)*

*Ichneumon appendigaster*: Linnaeus (1758) (original description) [sex unknown], deposited at LSUK, labels: “habitat in America.” Taxonomic history documented by Deans (2005).

## Description

*Body length.—*7.0–7.8 mm.

*Head.—*Median clypeal projection sharpness: blunt. Lower face texture: foveae absent. Median carina of lower face presence: absent. Malar space length: longer than 0.5 of compound eye height. Carinae laterally on frons presence: absent. Antennal rim shape: not raised laterally. Antennal shelf presence: present. Eye color: blue. Long setae (length *>*2× ocellus diameter) presence: absent. Mandibular teeth count: 3. Female scape length: greater than eye height. Female flagellum color pattern: monocolored. Female flagellum ventral sensillar patch spatial arrangement: F5–F11.

*Mesosoma.—*Shape of median area of pronotum: not recurved. Sulcus delimiting pronotal lobe presence: present. Pronotal lobe carina presence: absent. Anteromedian carina of the prosternum presence: absent. Mesoscutal humeral sulcus continuity: discontinuous. Notaulus shape: sigmoid. Notaulus lateral margins alignment: parallel. Median mesoscutal area shape: not prominent relative to lateral mesoscutal area. Scutoscutellar suture structure: not foveate. Ventro-lateral region of mesosoma texture: foveate. Anterolateral mesopectal projection 2d shape: scalene triangular. Speculum presence: absent. Epicnemium sculpture: smooth. Epicnemial carina shape: convex medially. Ventral margin of mesopectus length: shorter than ventral margin of metapectus length. Metapleural sulcus position: horizontal. Posterior margin of the propodeum ventrally of the propodeal foramen lateral view shape: convex. Gastral scrobe conspicuousness: inconspicuous. Lateral carina of gastral scrobe presence: absent. Submedian propodeal projection presence: absent. Nucha presence: absent. Female metatibial spines presence: absent. 1M length versus 1CUb length: 1M is distinctly longer than 1CUb. Distal part of 4RS shape: straight or arched proximally. Costal cell coloration: brown in the distal one-tenth.

*Metasoma.—*Petiole texture: smooth. Petiole pilosity: dense. Lateroventral carina of the petiole presence: present. Setiferous patch on dorsal region of abdominal terga 4–7 in female presence: present.

### Material Examined

Syntype male: {no locality label} LINN 2719 (LSUK). Syntype female: {no locality label} LINN 2720 (LSUK). Other material: FRANCE: New Caledonia: one female. NCSU 49532 (PSUC). USA: TX: Brazos Co.: one female. NCSU 23566 (PSUC).

### Remarks

The two putative syntype specimens deposited at LSUK are not conspecific. The male specimen (LINN 2719) is morphologically consistent with concept of *E. appendigaster* used in the majority of literature, whereas the female specimen (LINN 2720) is easily diagnosed, based on surface sculpture, mesosoma shape, appendage color, and wing venation, as *Prosevania fuscipes* (Illiger 1807). We wait to designate a lectotype, however, until these specimens can be observed directly.

Szepligetella deercreeki *Deans and Mikó sp. nov. (Figs. 3a, 4a, 6e, 7e, f)*

*Diagnosis.—*Differs from all known *Szepligetella* species in the combination of the following character states: notaulus lateral margins alignment: diverging; median mesoscutal area shape: prominent relative to lateral mesoscutal area; setiferous patch on dorsal region of abdominal terga 4–7 in female presence: absent.

### Description

*Body length.—*5.3–6.6 mm.

*Head.—*Median clypeal projection sharpness: pointed. Lower face texture: foveate. Median carina of lower face presence: absent. Malar space length: shorter than 0.5 of compound eye height. Carinae laterally on frons presence: present. Antennal rim shape: raised laterally. Antennal shelf presence: absent. Eye color: gray-silver. Long setae (length *>*2× ocellus diameter) presence: absent. Mandibular teeth count: 4. Female scape length: equal to eye height. Female flagellum color pattern: monocolored. Female flagellum ventral sensillar patch spatial arrangement: F4–F11.

*Mesosoma.—*Shape of median area of pronotum: recurved. Sulcus delimiting pronotal lobe presence: absent. Pronotal lobe carina presence: present. Anteromedian carina of the prosternum presence: present. Mesoscutal humeral sulcus continuity: continuous. Notaulus shape: sigmoid. Notaulus lateral margins alignment: diverging. Median mesoscutal area shape: prominent relative to lateral mesoscutal area. Scutoscutellar suture structure: foveate. Ventro-lateral region of mesosoma texture: areolate. Anterolateral mesopectal projection 2d shape: isosceles triangular. Speculum presence: present. Epicnemium sculpture: wrinkled. Epicnemial carina shape: concave medially. Ventral margin of mesopectus length: longer than ventral margin of metapectus length. Metapleural sulcus position: vertical. Posterior margin of the propodeum ventrally of the propodeal foramen lateral view shape: straight. Gastral scrobe conspicuousness: conspicuous. Lateral carina of gastral scrobe presence: absent. Submedian propodeal projection presence: absent. Nucha presence: present. Female metatibial spines presence: present. 1M length versus 1CUb length: 1M is distinctly longer than 1CUb. Distal part of 4RS shape: straight or arched proximally. Costal cell coloration: brown in the distal one-tenth; brown in the distal one-half–two-third.

*Metasoma.—*Petiole texture: smooth. Petiole pilosity: sparse. Lateroventral carina of the petiole presence: absent. Setiferous patch on dorsal region of abdominal terga 4–7 in female presence: absent.

#### Material Examined

Holotype: (female): FRANCE: New Caledonia: NCSU 43287 (CNC). Paratypes (four males): FRANCE: New Caledonia: NCSU 41660 (CNC), NCSU 49529–49531 (BMNH).

#### Etymology

This species is named after Deer Creek High School in Edmond, Oklahoma, USA, for recently contributing to our understanding and appreciation of insects through poetry through the 2011 Hexapod Haiku Challenge, hosted by the NCSU Insect Museum.

Szepligetella irwini *Deans and Mikó sp. nov. (Figs. 3a, 4e, 6f)*

*Diagnosis.—Szepligetella irwini* and *S. levipetiolata* differ from all known *Szepligetella* species in the following character states: submedian propodeal projection presence: present. Female flagellum color pattern: banded; Carinae laterally on frons presence: present.

*Szepligetella irwini* differs from *S. levipetiolata* in the following character states: long setae (length *>*2× ocellus diameter) presence: present; female metatibial spines presence: absent.

### Description

*Body length.—*6.5–6.8 mm.

*Head.—*Median clypeal projection sharpness: pointed. Lower face texture: foveae absent. Median carina of lower face presence: present. Malar space length: shorter than 0.5 of compound eye height. Carinae laterally on frons presence: present. Antennal rim shape: raised laterally. Antennal shelf presence: absent. Eye color: gray-silver. Long setae (length *>*2× ocellus diameter) presence: present. Mandibular teeth count: 4. Female scape length: greater than eye height. Female flagellum color pattern: banded. Female flagellum ventral sensillar patch spatial arrangement: F6–F11.

*Mesosoma.—*Shape of median area of pronotum: recurved. Sulcus delimiting pronotal lobe presence: absent. Pronotal lobe carina presence: present. Anteromedian carina of the prosternum presence: present. Mesoscutal humeral sulcus continuity: continuous. Notaulus shape: sigmoid. Notaulus lateral margins alignment: parallel. Median mesoscutal area shape: not prominent relative to lateral mesoscutal area. Scutoscutellar suture structure: foveate. Ventro-lateral region of mesosoma texture: areolate. Anterolateral mesopectal projection 2d shape: isosceles triangular. Speculum presence: present. Epicnemium sculpture: wrinkled. Epicnemial carina shape: concave medially. Ventral margin of mesopectus length: longer than ventral margin of metapectus length. Metapleural sulcus position: vertical. Posterior margin of the propodeum ventrally of the propodeal foramen lateral view shape: convex. Gastral scrobe conspicuousness: inconspicuous. Lateral carina of gastral scrobe presence: absent. Submedian propodeal projection presence: present. Nucha presence: present. Female metatibial spines presence: absent. 1M length versus 1CUb length: 1M is distinctly longer than 1CUb. Distal part of 4RS shape: straight or arched proximally. Costal cell coloration: brown in the distal one-half–two-third.

*Metasoma.—*Petiole texture: smooth. Petiole pilosity: sparse. Lateroventral carina of the petiole presence: absent. Setiferous patch on dorsal region of abdominal terga 4–7 in female presence: absent.

#### Material Examined

Holotype female: FRANCE: New Caledonia: NCSU 41664 (MNHN). Paratype female: FRANCE: New Caledonia: 44113 (INHS).

#### Etymology

The species name refers to the collector of the type specimens, M. E. Irwin.

**Figure 8:**
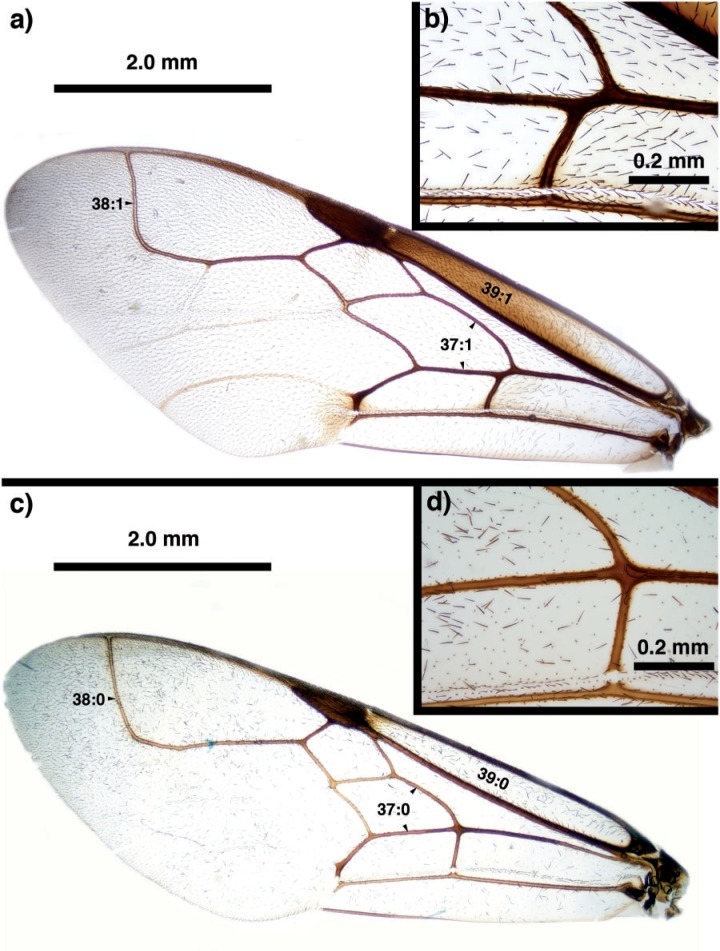
Brightfield images of the fore wing in New Caledonian *Szepligetella* species. (a, b) *Szepligetella levipetiolata* (Turner 1919). (c, d) *Szepligetella sericea* (Cameron 1883).

**Figure 9:**
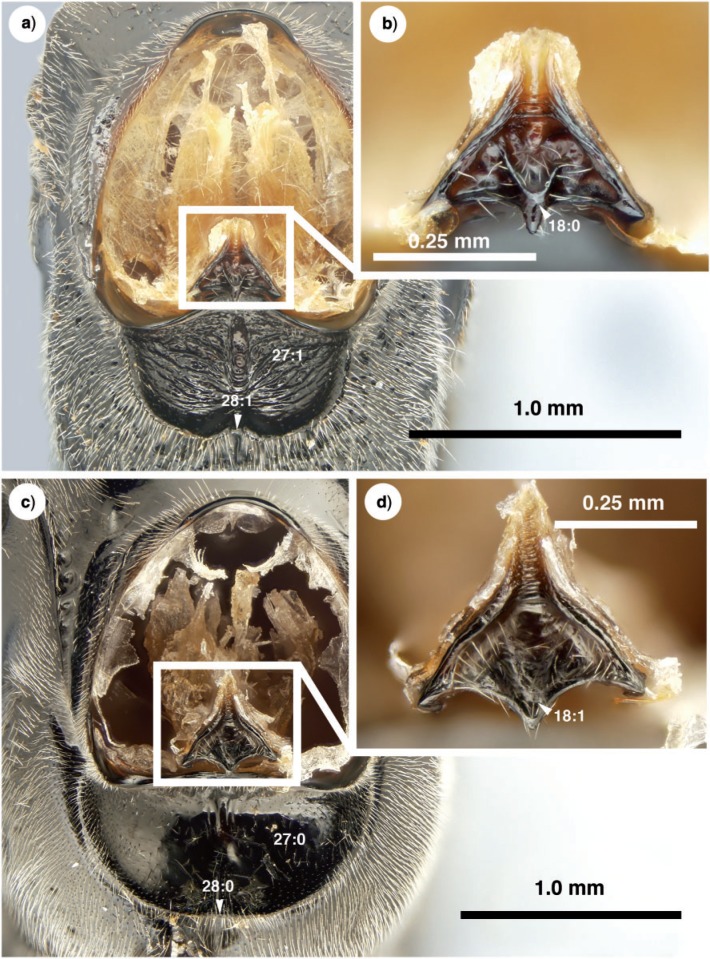
Brightfield images of the mesosoma and prosternum in New Caledonian Evaniidae, anterior view (propleura removed). a) *Szepligetella levipetiolata* (Turner 1919), mesosoma. b) *Szepligetella levipetiolata* (Turner 1919), prosternum. c) *Evania appendigaster* (Linneaus 1758), mesosoma. d) *Evania appendigaster* (Linneaus 1758), prosternum.

Szepligetella levipetiolata *(Turner 1919) (Figs. 4c, 5b, d, 6a, b, 7a, b, 8a, 9a, b)*

*Evania levipetiolata* Turner (1919) (original description).

Taxonomic history documented by Deans (2005).

### Description

*Body length.—*4.6–8.0 mm.

*Head.—*Median clypeal projection sharpness: pointed. Lower face texture: foveae absent. Median carina of lower face presence: present. Malar space length: shorter than 0.5 of compound eye height. Carinae laterally on frons presence: present. Antennal rim shape: raised laterally. Antennal shelf presence: absent. Eye color: gray-silver. Long setae (length *>*2× ocellus diameter) presence: absent. Mandibular teeth count: 4. Female scape length: greater than eye height. Female flagellum color pattern: banded. Female flagellum ventral sensillar patch spatial arrangement: F6–F11.

*Mesosoma.—*Shape of median area of pronotum: recurved. Sulcus delimiting pronotal lobe presence: absent. Pronotal lobe carina presence: present. Anteromedian carina of the prosternum presence: present. Mesoscutal humeral sulcus continuity: continuous. Notaulus shape: sigmoid. Notaulus lateral margins alignment: parallel. Median mesoscutal area shape: not prominent relative to lateral mesoscutal area. Scutoscutellar suture structure: foveate. Ventro-lateral region of mesosoma texture: areolate. Anterolateral mesopectal projection 2d shape: isosceles triangular. Speculum presence: present. Epicnemium sculpture: wrinkled. Epicnemial carina shape: concave medially. Ventral margin of mesopectus length: longer than ventral margin of metapectus length. Metapleural sulcus position: vertical. Posterior margin of the propodeum ventrally of the propodeal foramen lateral view shape: straight. Gastral scrobe conspicuousness: inconspicuous. Lateral carina of gastral scrobe presence: absent. Submedian propodeal projection presence: present. Nucha presence: present. Female metatibial spines presence: absent; present. 1M length versus 1CUb length: 1M is distinctly longer than 1CUb. Distal part of 4RS shape: straight or arched proximally. Costal cell coloration: brown in the distal one-half–two-third.

*Metasoma.—*Petiole texture: smooth. Petiole pilosity: sparse. Lateroventral carina of the petiole presence: absent. Setiferous patch on dorsal region of abdominal terga 4–7 in female presence: present.

#### Material Examined

Holotype male: FRANCE: New Caledonia, B.M.TYPE.HYM. 3a.287 (BMNH). Other material: FRANCE: New Caledonia: 87 males, 1 female (NCSU 41656). NCSU 51293 (CAS); NCSU 34829, 34842, 34848, 34860, 41650, 41654, 41656, 44107, 44114, 44116, 44119, 44124, 51298 (PSUC); NCSU 33643, 41652–41653, 41655, 41657, 41661–41662, 41665–41666, 41669, 41897–41898, 44100, 44103, 44108, 44110–44112, 44115, 44117, 44120, 44122–44123, 44125–44126, 44129, 51297, 51299 (MNHN); NCSU 34828, 34830–34841, 34843–34847, 34849–34859, 34861–34865, 41658, 41668, 44101–44102, 44104–44106, 44109, 44127, 51296 (INHS); NCSU 51294–51295 (CNC).

Szepligetella sericea *(Cameron 1883) (Figs. 6d, 7d, 8c, d)*

*Evania sericea* Cameron (1883). Taxonomic history documented by Deans (2005).

*Evania impressa* Schletterer (1889), syn. nov. (junior subjective synonym). Taxonomic history documented by Deans (2005).

### Description

*Body length.—*5.5–9.1 mm.

*Head.—*Median clypeal projection sharpness: pointed. Lower face texture: foveate. Median carina of lower face presence: present. Malar space length: shorter than 0.5 of compound eye height. Carinae laterally on frons presence: present. Antennal rim shape: raised laterally. Antennal shelf presence: absent. Eye color: gray-silver. Long setae (length *>*2× ocellus diameter) presence: absent. Mandibular teeth count: 4. Female scape length: equal to eye height. Female flagellum color pattern: monocolored. Female flagellum ventral sensillar patch spatial arrangement: F4–F11.

*Mesosoma.—*Shape of median area of pronotum: recurved. Sulcus delimiting pronotal lobe presence: absent. Pronotal lobe carina presence: present. Anteromedian carina of the prosternum presence: present. Mesoscutal humeral sulcus continuity: continuous. Notaulus shape: falciform. Notaulus lateral margins alignment: parallel. Median mesoscutal area shape: not prominent relative to lateral mesoscutal area. Scutoscutellar suture structure: foveate. Ventro-lateral region of mesosoma texture: areolate. Anterolateral mesopectal projection 2d shape: isosceles triangular. Speculum presence: present. Epicnemium sculpture: wrinkled. Epicnemial carina shape: concave medially. Ventral margin of mesopectus length: longer than ventral margin of metapectus length. Metapleural sulcus position: vertical. Posterior margin of the propodeum ventrally of the propodeal foramen lateral view shape: straight. Gastral scrobe conspicuousness: conspicuous. Lateral carina of gastral scrobe presence: present. Submedian propodeal projection presence: absent. Nucha presence: present. Female metatibial spines presence: present. 1M length versus 1CUb length: equal. Distal part of 4RS shape: arched distally. Costal cell coloration: brown in the distal one-tenth.

*Metasoma.—*Petiole texture: furrowed. Petiole pilosity: sparse. Lateroventral carina of the petiole presence: absent. Setiferous patch on dorsal region of abdominal terga 4–7 in female presence: present.

#### Material Examined

*Evania sericea* Cameron (1883) only known type male: USA: Hawaii, B.M.TYPE.HYM. 3a.288 (BMNH). *Evania impressa* Schletterer (1889) syntype female: PHILIPPINES (ZMHB). Other syntypes, deposited at ZMUH, were destroyed during World War II. Other material: FIJI: three males, five females, one sex unknown. NCSU 51433–51436, 51438 (CAS); NCSU 51437, 51439 (USNM); NCSU 53081 (PSUC); NCSU 53082 (BPBM). FRANCE: French Polynesia: 36 males, 3 females. NCSU 49536–49544, 51421–51427, 51429–51432 (CAS); NCSU 49545, 51428 (PSUC); NCSU 49546–49547, 49551, 51396, 51398, 51400, 51442–51445, 51447–51452 (USNM); NCSU 51446 (USNM). INDONESIA: nine males, three females. NCSU 51394, 51402–51411 (CAS); NCSU 51420 (USNM). MALAYSIA: one female. NCSU 51440 (USNM). MARSHALL ISLANDS: seven males, two females. NCSU 51397, 51412–51419 (USNM). PHILIPPINES: one male. NCSU 51401 (USNM). USA: one male. NCSU 51395 (USNM). USA: Hawaii: one male, one female. NCSU 49554, 51393 (USNM). USA: Hawaii: Hawaii Co.: nine males. NCSU 34866, 49548–49549, 51386–51391 (USNM). USA: Hawaii: Honolulu Co.: two males, two females. NCSU 51385 (CAS); NCSU 49553, 51392, 51441 (USNM). USA: Hawaii: Maui Co.: one male, one female. NCSU 49552 (CAS); NCSU 49555 (PSUC). VANUATU: one female. NCSU 49550 (CAS).

### Comments on the Ensign Wasps of New Caledonia

There are likely *>*100 species of cockroaches on New Caledonia that construct egg cases (P. Grandcolas, *in litt.*) which could serve as “hosts” for ensign wasp larvae. Yet, we could only find two species of evaniid that are presumably native to the island and one introduced species, whose hosts are well known. The two native ensign wasp species could be (i) generalists that predate on numerous cockroach species (as has been discussed for other evaniids; see Deyrup and Atkinson 1993), (ii) cryptic species complexes that could not be distinguished during our study, and/or (iii) part of a larger fauna of microendemic species, most of which have yet to be collected and described. This revision hopefully catalyzes future efforts to elucidate the natural history of the New Caledonian ensign wasp fauna.

## Discussion

### Phenotype Annotation

Although semantic annotation of evolutionary character matrices has been initiated post hoc for some published studies (Dahdul et al. 2010a), this article along with Mullins et al. (2012) are the first publications to provide *both* new taxonomic descriptions *and* corresponding semantic data. We aim to take the first steps along the path outlined by Deans et al. (2012), toward the creation of computable and reusable phenotypic data as a product of taxonomic studies. The ontological annotation of 43 characters here, and 56 in Mullins et al. (2012), resulted in a rich, queryable OWL data set, reveals several challenges inherent in representing natural phenotypes in OWL, and suggests means to better facilitate the application of semantic technologies within systematics.

### SP Expressivity

A critical consideration for the application of semantic technology to phenotypic descriptions is how well a logical representation can express the meaning currently conveyed by NL. For example, creating logical statements that encapsulate the full meaning of the single NL statement “aedeagus apical column broad, and flattened apically, ending abruptly with rounded basal angle and narrow, hooked apical lobe, column extending more than half its length beyond penis valve, bending dorsally,” is a much larger challenge relative to the statement “gonocoxa orange” (both statements from Baptiste and Kimsey 2000). This will depend on the logical expressivity of the knowledge representation system being used (the types of statements it is possible to make), and also how straightforward it is for scientists to apply features of the language to their descriptions. We focus our approach on the OWL family of knowledge representation languages for several reasons. First, as the ontology language of the semantic web, OWL identifiers are URIs: global identifiers suitable for publishing and referencing data directly on the web. Second, OWL is a free standard with a formal description logic foundation; this has allowed the development of multiple freely available, compatible, automated reasoning systems, each with its own strengths and weaknesses. There is an active community of users developing tools for working with OWL. Finally, OWL is being used for many life sciences semantic applications, and the W3C (World Wide Web Consortium) has actively considered use cases from the life sciences community in the development of semantic web technologies, for example, through the Semantic Web Health Care and Life Sciences Interest Group (http://www.w3.org/blog/hcls/ last accessed May 13, 2013).

Mungall et al. (2007) provided an OWL model for phenotypes based on the EQ approach. They found that composing phenotypes as OWL class expressions works well, with minor exceptions, for a variety of phenotypes, promoting modular reuse of ontologies and rigorous reasoning. An application of the basic EQ model to more than 4600 characters from the phylogenetic systematic literature found phylogenetic characters to be very amenable to EQ description (Dahdul et al. 2010a). However, one challenge noted in that study was the difficulty of annotating the finer aspects of the quality portion of the EQ for many characters. This was addressed by selecting a predefined set of “upper” quality terms from the PATO ontology, which curators used to provide a “coarse granularity” of phenotype annotation. This coarse annotation accelerated curation but still provided useful classification of phylogenetic characters.

Building on these forays into EQ phenotype annotation, we found that the expressivity of OWL DL was adequate for most of our characters. The OWL expressions lack some of the subtlety possible in natural-language descriptions, but because we defined our characters with semantic annotation in mind (i.e., we forced ourselves to describe character states explicitly from the outset), EQ translation was generally straightforward. However, as described in the “Results” section, one class of characters, “relative measurements,” proved challenging to adequately describe using OWL. Five out of the 43 characters described here involved relative measurements. Although they constitute only a little more than 10% of the characters in this study, relative measurements are a common and important means for describing morphological changes in a size-independent manner. The OWL limitation stems from a requirement that class descriptions exhibit a tree-like structure, a factor in decidability (Motik et al. 2009). Although a semantic model of “instance-level” structure measurements of a particular organism can be adequately constructed using class and property assertions on a graph of OWL instance nodes, the general definition for a class of all organisms with a given relative measurement cannot be fully described in OWL 2 (Magka et al. 2012). Although our relative measurement annotations do not capture the full meaning intended by each character, we feel that even with these limitations they provide useful semantic context for the characters. The classes of structures involved (e.g., “metapectus” and “mesopectus”), and the quality being compared (e.g., “length”), are captured within the semantic annotation, facilitating useful ontology-driven queries over the characters. Development of better reasoning approaches for semantic data is an active area of research, and the tree-model restriction of OWL is being specifically addressed, as it is a limitation for other scientific descriptions such as that of chemical structures (Hastings et al. 2010). Future versions of OWL (or a related language) involving “description graphs” may allow much more complete representation of, and more powerful reasoning with, these characters (Magka et al. 2012).

With representational limitations in mind, we suggest that semantic annotation should be primarily considered as a means to *classify* NL phenotypic descriptions, rather than fully replace them. In contrast, Vogt et al. (2010) advocate direct representation of morphological data using RDF statements. It may be that such an approach, with the right tools, becomes feasible for “instance-level” description (above), whereas complete definition of character-state classes, as explored here, is less so. By reference to shared ontologies, both approaches become interoperable. Semantic annotations can describe the implications of a character state for denoted organisms as far as is feasible, allowing inference of implied facts and facilitating search through logical subsumption and semantic similarity approaches. Although semantic annotations as provided here may not represent every detail expressed in each character state, they should also not result in any unintended inferences.

Indeed, the ontological classification of characters in this study and in Mullins et al. (2012) allowed straightforward comparison of anatomical coverage of the phenotypic descriptions in each (Tables 2 and 3). Although these analyses are admittedly simplistic, as the number of available semantic data sets increases, more ambitious use cases will become feasible (Deans et al. 2012). Semantic similarity approaches (e.g., Washington et al. 2009) across Metazoa will be facilitated by further development of comprehensive multispecies anatomy ontologies such as Uberon (Mungall et al. 2012), which currently provides a coherent ontological structure, to varying degrees of granularity, for the anatomy of many animal groups including vertebrates, insects, and even echinoderms.

### Annotation Workflow

It is clear that widespread adoption of semantic methodologies within systematics will require development of tools that facilitate, rather than complicate, systematists' work. The initial approach demonstrated here requires some familiarity with both OWL and the Protégé application. Our cdao-protege plugin made navigation of characters and associated states within Protégé fairly straightforward; however, the mechanics of manually creating ontology classes for phenotypes and consistently creating all the required links to character states did prove challenging to nonexpert Protégé users. Even so, it is our experience that interested biologists can quickly comprehend and apply OWL class descriptions, particularly using the English-like Manchester Syntax. We have prototyped a fully integrated SP annotation interface directly within mx, building on the approach begun with the Phenex annotation application (Balhoff et al. 2010). However, we found that more experience with the composition of real annotation results, created in the unconstrained environment of Protégé, would be required to better assess the requirements of such a system for taxonomy. These data sets will provide the basis for further work in that area.

Direct creation of phenotypic class expressions within Protégé provides the annotator a maximum of freedom to express the meaning of the phenotype as closely as possible; with this freedom comes the possibility that the annotator may create unintended logical inferences, whether through mistakes or lack of ontology expertise. An alternative approach we plan to explore further is a simpler ontology “tagging,” wherein high-throughput text-processing systems, for example, Textpresso (Müller et al. 2004) or CharaParser (Cui 2010, 2012), can be used to more quickly identify terms within phenotype descriptions that can be matched to ontology classes. This approach would support basic query answering over character descriptions using anatomy and quality ontologies, but, by removing the “internal” semantics of the phenotype, would eliminate some use cases requiring more sophisticated inferences about organisms, such as analysis of presence/absence of structures across evolution. For Hymenoptera specifically, we have provided a solution that lies between the complexity of producing SPs and traditional treatments alone. Hymenopterists can use the “analyze” functionality at the HAO portal (http://portal.hymao.org last accessed May 13, 2013) to compare their descriptive text against the HAO and to return ontology concept URIs (Seltmann et al. 2012).

Either approach, careful SP description or simple semantic tagging, requires a well-developed multispecies anatomy ontology for maximum utility. The importance of expert morphologists has never been greater in this regard (Dahdul et al. 2010b; Yoder et al. 2010). Community efforts are underway to coordinate development of such ontologies (see the Phenotype Research Coordination Network, http://www.phenotypercn.org/ last accessed May 13, 2013).

### Dissemination

As the ontology language for the Semantic Web, OWL not only provides an ontological reasoning framework but also a means to publish our descriptions as RDF, contributing to the emerging universe of Linked Open Data (Bizer et al. 2009). The use of shared, community-driven ontologies, containing standard concepts with global identifiers, promotes integration across data sets (Washington et al. 2009; Mungall et al. 2010). Beyond ontology concepts, RDF allows us to provide every data element with its own global URI, including characters, states, and OTUs. Publishing descriptive data in this way on the Semantic Web should facilitate explicit reuse of characters across studies: anyone can code another taxon for published characters in a way that can be seamlessly integrated with existing data. As character-centric semantic datastores such as the Phenoscape Knowledgebase (Mabee et al. 2012) expand in scope, richly annotated taxonomic descriptions will be ripe for inclusion.

## Supplementary Material

Data files and/or other supplementary information related to this paper have been deposited at Dryad under http://dx.doi.org/10.5061/dryad.2gd84.

## Funding

This material is based on work supported by the National Science Foundation (grant numbers DBI-0850223, DEB-0842289, DEB-0956049, and EF-0905606); and the National Evolutionary Synthesis Center and benefited from discussions initiated through the Phenotype Research Coordination Network (in part). Any opinions, findings, and conclusions or recommendations expressed in this material are those of the authors and do not necessarily reflect the views of the National Science Foundation.
